# Insect-Based Feed Ingredients for Aquaculture: A Case Study for Their Acceptance in Greece

**DOI:** 10.3390/insects12070586

**Published:** 2021-06-28

**Authors:** Christos I. Rumbos, Eleni Mente, Ioannis T. Karapanagiotidis, Georgios Vlontzos, Christos G. Athanassiou

**Affiliations:** 1 Laboratory of Entomology and Agricultural Zoology, Department of Agriculture, Crop Production and Rural Environment, School of Agricultural Sciences, University of Thessaly, Phytokou Str., N. Ionia, 38446 Volos, Greece; athanassiou@uth.gr; 2 Department of Ichthyology and Aquatic Environment, School of Agricultural Sciences, University of Thessaly, Phytokou Str., N. Ionia, 38446 Volos, Greece; emente@uth.gr (E.M.); ikarapan@uth.gr (I.T.K.); 3 Laboratory of Agricultural Economy and Consumer’s Behaviour, Department of Agriculture, Crop Production and Rural Environment, School of Agricultural Sciences, University of Thessaly, Phytokou Str., N. Ionia, 38446 Volos, Greece; gvlontzos@uth.gr

**Keywords:** aquaculture, insect-based aquafeeds, survey, public perceptions

## Abstract

**Simple Summary:**

Since 2017, insects can be used as ingredients in aquafeeds in the EU. However, insect-based aquafeeds are still not broadly accepted by European aquaculture companies. Understanding the beliefs of people associated with the aquaculture sector on the use of insect-based fish diets could assist their adoption. In the present study, we ran a survey among the participants of an aquaculture conference held in Greece, in order to ask them what they think regarding the inclusion of insect meal in aquafeeds. Furthermore, we inquired nine Greek aquaculture and aquafeed companies about this issue. Greece is among the largest farmed fish producers in the EU; however, there are currently no data available on the acceptance of insect-based aquafeeds in Greece. Based on our results, the majority of the respondents were aware and in favor of the inclusion of insects in aquafeeds, mainly due to their potential to lower fishing pressure on wild fish stocks used for fishmeal production and enhance the ecological footprint and sustainability of aquaculture. Moreover, six out of nine companies were favorably disposed towards the use of insects in fish diets and four of them were willing to produce or use such diets. Further studies are warranted towards this direction.

**Abstract:**

Although the inclusion of insects in fish diets is officially allowed in the EU since 2017, insect-based aquafeeds have not been widely adopted by the European aquaculture sector. In order to investigate the perceptions related with adoption trends, it is critical to explore the beliefs of people associated with the aquaculture sector on the use of insects in farmed fish diets. A survey was conducted among 228 participants of an aquaculture conference to explore their perceptions on the inclusion of insect meal in fish diets. Additionally, we investigated the attitudes of nine companies operating in the aquaculture and aquafeed sector in Greece that attended the conference towards this direction. The findings of the conference survey provide evidence that there is a wide-range awareness and acceptance regarding the use of insect-based feeds in farmed fish diets among the respondents. This is mainly driven by the expectations for the decline in fishing pressure on wild fish stocks, the reduction of the ecological footprint and the enhancement of the sustainability of the aquaculture sector. The results of the stakeholder survey show that six out of the nine companies that participated in the survey are favorably disposed towards the use of insect-based feeds. Specifically, four of them stated that they would produce or use aquafeeds based on insects. However, the results highlight the need for further research on the implementation of the wider adoption of insect-based feeds in aquaculture. The present study provides some first insights into the use of insect-based aquafeeds in Greece, for which there are no data available.

## 1. Introduction

The world aquaculture production continues to increase with high annual growth rates [1]. According to the Food and Agriculture Organization (FAO) of the United Nations, global aquaculture production of farmed food fish in 2018 reached 82 million tons with a value of USD 400 billion, representing around half of the total world fish production for human consumption [1]. Greece is among the largest farmed fish producers in the EU [1] with more than 300 fish farms with an annual production in 2019 of around 125,000 tons of fish [2]. The aquaculture industry is an important pillar of the Greek agricultural production, since marine fish is the top animal product exported contributing about 11% to the total national agricultural exports [3]. The gilthead sea bream, *Sparus aurata* L., and the European sea bass, *Dicentrarchus labrax* L., are the two main farmed species in Greek fish farms, with a total production of around 117,000 tons, from which approximately 80% is exported [2]. Particularly for these two species, Greece is one of the major world suppliers, as it provides 58% of the total sales in EU and 24% of the sales worldwide [2].

A consequence of the rising aquaculture production globally is the increasing demand for industrially compound aquafeeds, in particular fishmeal, which constitutes the main protein source of farmed fish diets [4,5]. Hence, the fact that the global fishmeal production is stable for the last two decades causes the fishmeal price to rise, which, in turn, generates an increase in the total cost of aquaculture production [6,7]. Therefore, the aquaculture and aquafeed industries are urgently in need of efficient and viable alternatives to fishmeal for aquaculture feeds [8,9]. During the past decades, the sector has proceeded to major reductions of fishmeal in aquafeeds using plant proteins [10,11] and most recently using land animal proteins [12,13]. However, as aquaculture develops and intensifies, significant amounts of this limited natural resource is being used in aquafeeds and, thus, fishmeal replacement is still of a high priority.

One of the promising and sustainable alternative protein sources to fishmeal for use in aquafeeds are insects [14,15,16]. The potential of insects as a source of nutrients for food and feed has been identified early enough [17]. Insects are highly nutritious, convert feed efficiently to body mass, can be grown on organic side-streams and agricultural by-products, whereas their rearing is described by low water and land requirements and reduced gas emissions [18,19]. The use of insect meals as a fishmeal replacement has been successfully evaluated for a range of fish species, such as the European sea bass [20]; trout, *Oncorhynchus mykiss* (Walbaum) [21,22,23]; Nile tilapia, *Oreochromis niloticus* (L.) [24,25]; gilthead seabream [26,27]; the Siberian sturgeon, *Acipenser baerii* Brandt [28]; the clownfish, *Amphiprion ocellaris* Cuvier [29]; or the zebrafish, *Danio rerio* (Hamilton) [30], and shrimps [31]. By the EU Regulation 2017/893 in 1 July 2017, the exploitation of insect protein in fish diets has been allowed in Europe [32]. Specifically, the EU Regulation authorizes the use of processed animal protein derived from seven insect species for aquaculture, namely the black soldier fly, *Hermetia illucens* (L.) (Diptera: Stratiomyidae); the common housefly, *Musca domestica* L. (Diptera: Muscidae); the lesser mealworm, *Alphitobius diaperinus* (Panzer) (Coleoptera: Tenebrionidae); the yellow mealworm, *Tenebrio molitor* L. (Coleoptera: Tenebrionidae); and three Gryllidae (Orthoptera): the house cricket, *Acheta domesticus* (L.); the banded cricket, *Gryllodes sigillatus* (Walker); and the field cricket, *Gryllus assimilis* (F.). This is particularly important for the European countries, with well-developed aquaculture industry, i.e., Norway, Spain, UK, France, Italy and Greece, since the reliance on fishmeal-based aquafeeds has greatly contributed to the increase in production cost. Apart from aquafeeds, the suitability of insect meals as ingredient of poultry diets has been documented by several studies [33,34,35,36], whereas it is believed that soon the EU will authorize the use of insects for the poultry and swine industry.

Interestingly, although a lot of research has been conducted in the last years on the potential of insects as a nutrient source for farmed fish, information on the views of people associated in several ways with the aquaculture sector on the use of insects in aquafeeds is limited. Most studies exploring the public opinion regarding the use of insects as food or feed have investigated the consumers’ perceptions towards insect-based foods [37,38]. Regarding the acceptance of insects as animal feed in general and in particular as aquafeed, information is limited, and usually area or case-specific [39,40,41,42,43,44,45]. One of the few recently published studies explored the attitude of farmers, agriculture sector stakeholders and citizens towards insect-based animal feed, as well as their willingness and readiness to accept them and reported in general favorable attitudes towards the use of insects in animal feed [39]. More recently, the perceptions of Spanish customers for the use of insect meals in aquafeeds was investigated and their willingness to pay a premium for gilthead seabream fed with insect meal compared with fish produced with the conventional feeding systems was demonstrated [42]. A similar study in the UK for the Scottish Atlantic Salmon, *Salmo salar* L., reported favorable attitudes of consumers, as well as stakeholders of the salmon farming sector, towards the use of insect meals [41]. However, research on the public opinion on the exploitation of insect-based feeds in aquaculture is far from being exhaustive, and requires additional data for a wider range of target audiences and chain scenarios. At the same time, the data are still limited for many of the European countries with well-developed aquaculture industry, especially in the case of fish producers and related stakeholders. Particularly for Greece, currently there are no data available regarding the acceptance of the use of insects in fish diets.

Although quite a bit of time has passed since the EU gave the “green light” for the use of insect protein in the diets of farmed fish, insect-based aquafeeds are very far from being a common practice for the European aquaculture and aquafeed companies. In order to understand the main reasons for this, and to facilitate the future adoption of insect-based feeds by the aquaculture sector, it is critical to shed light upon the perceptions and beliefs of people and companies associated in several ways with the aquaculture sector about the use of insect protein in aquafeeds. In this context, the objective of the present study is to provide first insights into the level of acceptance of using insects in the diets of farmed fish in Greece. Therefore, a survey was conducted among the Greek participants of an aquaculture conference to explore their perceptions on the inclusion of insect meal in fish diets. Additionally, we investigated the attitudes of Greek companies (aquaculture and aquafeed companies) towards the production and use of insect-based feeds in aquaculture. This is the first survey on the acceptance of insect-based aquafeeds in Greece.

## 2. Materials and Methods

### 2.1. Aquaculture Conference Attendees Survey

The survey was undertaken during the 3rd International Congress on Applied Ichthyology and Aquatic Environment (HydroMediT 2018), organized in Volos, Central Greece, between 8 and 11 November 2018. HydroMediT, an international meeting of Applied Ichthyology and Aquatic Environment, is organized biannually and covers all main areas of aquaculture, fisheries and aquatic products. Greek participants of the HydroMediT 2018 conference were invited to complete a hard copy questionnaire on the use of insect proteins in aquafeeds at the stand of the University of Thessaly in the conference hall. In most of the cases, it did not take more than 10 min for the respondents to complete the questionnaire. During the four days of the conference, a total of 228 attendees were voluntarily recruited to take part in the survey. The sample included 158 undergraduate and postgraduate university students of various departments of Ichthyology and Aquatic Environment, 39 academics and researchers from the field of aquaculture, 7 public sector employees (government, public authorities, etc.) and 23 employees of companies from the aquaculture and aquafeed sector. Regarding the latter, they were asked to provide their personal attitude and perception on the topic.

The questionnaire distributed to the participants of the conference consisted of two sections. In the first section, participants were asked to provide socio-demographic data (gender, age, household income and education level). The second section inquired at first about the fish consumption frequency of the respondent. Then, participants were asked whether they were aware of the potential of insects as feed ingredients in aquaculture. Additionally, they were asked to state their level of agreement towards the idea of rearing insects for use as aquafeed ingredients on a five-point Likert scale with response categories ‘1 = strongly disagree’, ‘2 = disagree’, ‘3 = neutral’, ‘4 = agree’, ‘5 = strongly agree’. “Neutral” denoted that respondents had neither a positive nor a negative attitude, whereas they were also given the choice of “I don’t know”, as a possible answer option. Furthermore, participants were asked about their willingness to consume farmed fish fed on diets based on insects. Based on their response on this particular question, respondents were prompted to provide the main reasons for their answer. For instance, the participants who answered “Yes” and were willing to consume farmed insect-fed fish were asked to state on the previously described Likert scale to which extent their choice was dictated by (1) the lower fish price, their belief that the use of insects in aquafeeds helps (2) to reduce the reliance of aquaculture on fishmeal, (3) to enhance aquaculture sustainability, (4) to reduce the ecological footprint of aquaculture and (5) to lower the fishing pressure on wild fish stocks, through the reduction of the fishmeal used in aquaculture and (6) the better fish nutritional value. Similarly, participants who answered “No” to the consumption of insect-fed fish were prompted to highlight the main reasons for their response. Namely, using the above Likert scale, participants were asked to state whether their negative perception regarding insect-fed farmed fish was related to (1) their higher price, (2) their organoleptic characteristics (concerns about their taste, odor, etc.) (3) their quality and nutritional value, (4) the concerns about their safety and hygiene (e.g., potential microbial or allergy risks), (5) the negative impact on human health or (6) simply because they do not like the idea. Finally, participants who did not state a preference in favor or against the consumption of insect-fed fish were prompted to indicate which pieces of information (e.g., price, quality and nutritional value, organoleptic characteristics, food safety, hygiene and allergies, etc.) could be proved decisive for them to shape an opinion on this subject. In all cases, respondents were allowed to choose only one answer in each question.

### 2.2. Stakeholder Survey

In addition to the previous study, a second survey was conducted to investigate stakeholders’ awareness and attitudes towards the use of insect-derived materials in aquafeeds. Nine companies, operating in the aquaculture and aquafeed sector in Greece, took part in this survey. These companies participated in the aforementioned HydroMediT 2018 conference and represent approximately one quarter of the aquaculture sector and more than three quarters of the aquafeed sector in Greece, in terms of production volumes. A questionnaire was distributed shortly after the conference by email to the companies and their answers were received in a timely manner. In the first part of the questionnaire, general information regarding the company was requested, [name of the company, type (aquaculture or aquafeed business) and size (number of employees) of the company, its main products (aquafeeds, fish species), the annual production capacity for each product, its target market (national, European, world, etc.), and the age of the company]. In the second part, the questionnaire inquired about the awareness of the potential of using insects as an ingredient in fish feed in aquaculture, and the general attitude of the participated companies regarding this using a Likert scale. The answers ranged among ‘1 = strongly disagree’, ‘2 = disagree’, ‘3 = neutral’, ‘4 = agree’ and ‘5 = strongly agree’. Consequently, the participating companies were requested to state whether they would use or produce aquafeeds based on insects, indicating their response on a five-point Likert scale (‘1 = definitely no’, ‘2 = no’, ‘3 = neutral’, ‘4 = yes’, and ‘5 = definitely yes’). As previously explained, “neutral” denoted that companies had neither a positive nor a negative attitude, whereas they were also given the choice of “I don’t know”, as a possible answer option. Based on their response on this particular question, the companies were asked to justify their answer and provide the main reasons for their response. The companies in favor of using or producing insect-based aquafeeds, answering “yes” or “definitely yes” to the previous question, were asked whether their response was influenced by (1) the lower production cost, (2) the improved quality of the final products, the positive impact on (3) their competitiveness, (4) their innovation profile, (5) their sustainability profile, (6) their bargaining position and (7) the environmental footprint of the company. Those companies who were negative regarding the use or production of insect-based aquafeeds, answering “no” or “definitely no”, were asked to state to which degree their answer was due to their concerns about (1) potential reduced consumer acceptance of their products, (2) the increase in the production cost, (3) the reduced fish growth, (4) the concerns about the lower aquafeeds/fish quality and (5) the product safety or the doubts about potential benefits on (6) the enhancement of their sustainability or (7) the reduction of the environmental footprint of the company. Finally, those who had a neutral opinion or answered “I don’t know” regarding the use or production of insect-based feeds for farmed fish were requested to state on what issues they would like to have information in order to have a more informed view on this issue, e.g., the effect of the use of insects on (1) their product price, (2) the customer acceptance, (3) the legislative framework, the impact of the use of aquafeeds on (4) fish organoleptic characteristics, (5) fish growth and health and (6) product safety, as well as (7) on the extent that these practices help improve the sustainability and environmental profile of the company. For all participants asked to provide their views in favor or against the use or production of insect-based aquafeeds by their company, the responses were in a five-point Likert scale (‘1’ to ‘5’ from ‘strongly disagree’ to ‘strongly agree’, as well as ‘I don’t know’).

### 2.3. Statistical Analyses

Data analyses included descriptive statistics (means, frequencies, percentages). Moreover, the Chi-square test of independence (χ^2^) was performed to determine if there was a significant relationship between two nominal variables, i.e., the socio-demographic characteristics of the respondents (age, gender, household income and education level) and their responses to the survey. Mean attribute perception scores were compared with a test value (3), corresponding to “neutral perception”, using a one-sample *t*-test. For *p* < 0.05, the null hypothesis (H0), assuming there was no difference between the true mean and the test value, was rejected. For the company participants there was no statistical inference due to the small sample size. All analyses were performed using SPSS Statistics 26.0 (SPSS Inc., Chicago, IL, USA).

## 3. Results

### 3.1. Socio-Demographic Data

The socio-demographic characteristics of the respondents are presented in Table 1, where it is indicated that both genders were equally represented in our survey (51.3% females and 48.7% males). The participants’ age groups are between 17 and 72 years. However, the age distribution of the sample is skewed towards young ages. Specifically, the proportion of those that are younger than 30 years represent more than two thirds of the total sample (70.2%), while participants aged between 30 and 40, 40 and 50 and those older than 50 years old represent 11.0, 11.4 and 7.5% of the total sample, respectively. The respondents are students (69.3%), academics and researchers in the field of aquaculture (17.1%), staff of aquaculture or aquafeed companies (10.1%) and staff of the public sector (3.1%). Roughly, one half of the participants (45.2%) stated that they earn annually less than $20,000, 22.4% earn between $20,000 and 30,000, and 24.1% earn more than $30,000. This is in accordance with the average household income per capita in Greece, which is around $17,700 a year [46]. However, it should also be taken into account that the majority of the participants of the present survey were students, which are usually a low-income group. Finally, 32.2% hold a post-graduate degree, 21.5% a University degree, 16.2% are graduates of a college or a technical school and 29.8% are high school leavers.

### 3.2. Personal Attitudes towards the Use of Insects in Aquafeeds

Based on our results, the vast majority of the participants (64.0%) eat fish often (1–2 times per week), while the remainder eat fish 1–2 times per month (24.1%) or even more rarely (11.8%). The frequency of fish consumption is statistically affected by age (χ^2^ = 16.924, df = 8, *p* = 0.031). Older respondents eat more fish compared with their younger counterparts. Similarly, students consume significantly less frequently fish compared with the other groups (academics, stakeholders, etc.) (χ^2^ = 19.717, df = 10, *p* = 0.032).

**Figure 1:**
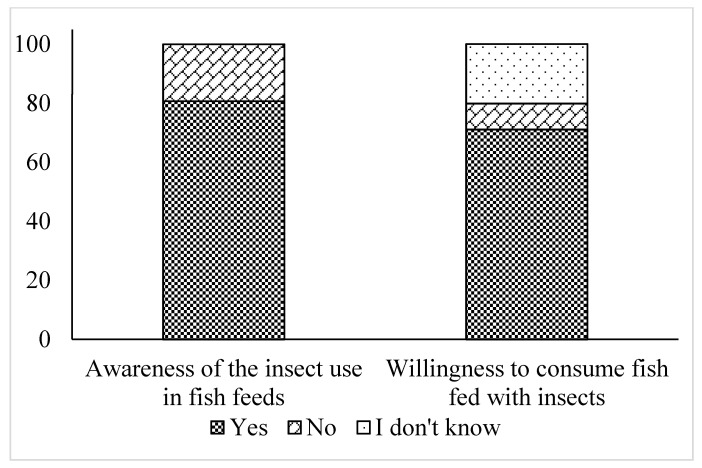
Awareness of the insect use in fish feeds and willingness to consume fish fed with insects (n = 228).

Regarding the level of awareness towards the use of insects as an ingredient in fish feeds in aquaculture, we found that 80.7% of the participants are informed about the potential of insect-based aquafeeds (Figure 1). However, there are statistically no gender effects (χ^2^ = 2.203, df = 1, *p* = 0.138), age (χ^2^ = 8.817, df = 4, *p* = 0.066), income effects (χ^2^ = 8.084, df = 9, *p* = 0.526) or educational level effects (χ^2^ = 3.906, df = 4, *p* = 0.419) on the awareness level. Among all groups of participants, the students are significantly less informed about the use of insects in aquafeeds (χ^2^ = 12.352, df = 5, *p* = 0.030).

The attitude towards the use of insects as feed for the rearing of farmed fish was favorable. The majority of the participants stated that they “agree” (45.2%) or “strongly agree” (22.4%) with this, whereas only 5.7% indicate a negative attitude, “disagreeing” (4.8%) or “strongly disagreeing” (0.9%) with this perspective. A considerable proportion of the respondents (21.9%) stated that they neither agree nor disagree, with the use of insects in aquafeeds, and only 4.8% stated that they do not have a clear view on this issue and need further information. Interestingly, male respondents are less inclined to accept the idea of rearing insects for use as an ingredient for fish feed, as compared with females (χ^2^ = 14.715, df = 5, *p* = 0.012). Moreover, 71.1% of the respondents’ stated that they would consume fish fed with insect-based feeds, while only 8.8% exhibited a negative attitude towards this direction. This result is not statistically affected by the socio-demographic characteristics. Finally, 20.2% of the participants indicated that they do not know whether they would consume fish fed with insects due to the lack of information on the topic.

**Figure 2:**
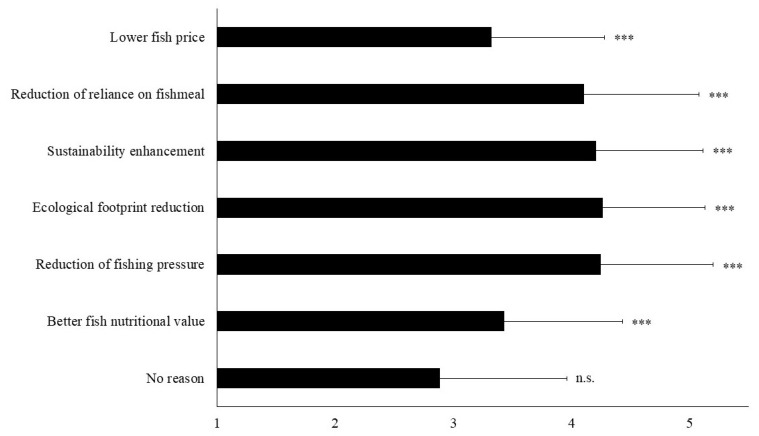
Mean attribute perception scores (± Standard Deviation) among participants willing to consume fish fed with insects for the main reasons that dictate their attitude. Answers were given on a five-point Likert scale with response categories ‘1 = strongly disagree’, ‘2 = disagree’, ‘3 = neutral’, ‘4 = agree’, ‘5 = strongly agree’ (n = 162). Asterisks denote statistical differences (*** *p* < 0.001) after an one-sample t-test with test value = 3; n.s.—not significant.

Figure 2 shows the ranking in terms of scores of the reasons stated by the participants as affecting their positive attitude towards the consumption of fish fed with insect meal. The enhancement of the ecological footprint of aquaculture has the highest score (mean score on the five-point Likert scale (Ls score = 4.27; t = 18.2, *p* < 0.001), followed by the decline in fishing pressure on wild fish stocks (Ls score = 4.25; t = 16.4, *p* < 0.001) and the aquaculture sustainability enhancement (Ls score = 4.21; t = 16.8, *p* < 0.001). The reduction of the reliance of the aquaculture sector on fishmeal (Ls score = 4.11; t = 14.0, *p* < 0.001) turns out to be also important. In contrast, the fish price Ls score was only 3.32 (t = 4.1, *p* < 0.001) and the fish nutritional value score only 3.43 (t = 5.1, *p* < 0.001). Since all the above mentioned scores are significantly different from the test value 3.00 that corresponds to a neutral perception, the null hypothesis that the mean scores were equal to three was in all cases rejected.

**Figure 3:**
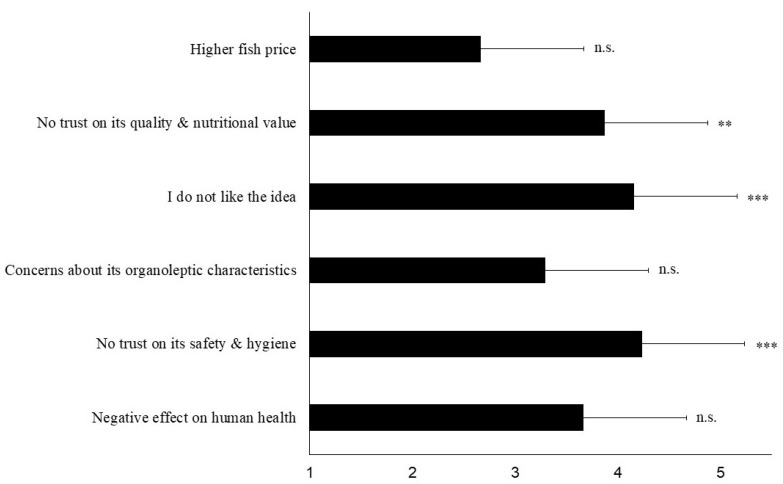
Mean attribute perception scores (±Standard Deviation) among participants not willing to consume fish fed with insects for the main reasons that dictate their attitude. Answers were given on a five-point Likert scale with response categories ‘1 = strongly disagree’, ‘2 = disagree’, ‘3 = neutral’, ‘4 = agree’, ‘5 = strongly agree’ (n = 20). Asterisks denote statistical differences (** *p* < 0.01, *** *p* < 0.001) after an one-sample t-test with test value = 3; n.s.— not significant.

Figure 3 illustrates the mean attribute perception scores among participants not willing to consume fish fed with insects for the main reasons that dictate their attitude. The most important was the negative perception for the hygiene and safety of insect-fed fish (Ls score = 4.24; t = 6.1, *p* < 0.001). At the same time, being repulsed by the idea of fish being fed with insects Ls score was 4.16 (t = 4.7, *p* < 0.001), and not trusting the quality and nutritional value of the insect-fed fish score was 3.87 (t = 3.1, *p* = 0.008). Concerns about the potential negative effects on human health scores 3.67 (t = 1.7, *p* = 0.116), while the respective figure for fish organoleptic characteristics (e.g., odor, taste, etc.) was 3.29 (t = 0.9, *p* = 0.369), followed by the potentially higher fish price (Ls score = 2.67; t = −1.05, *p* = 0.313). Nevertheless, the latter three reasons do not weight significantly in the participants’ perception for insect-fed farmed fish.

**Figure 4:**
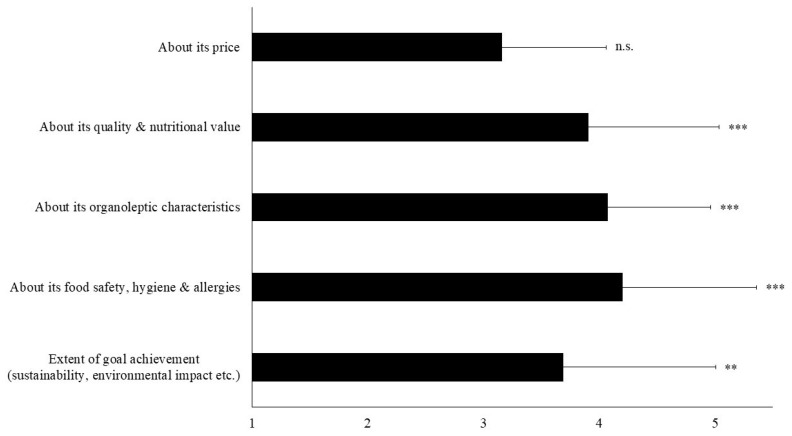
Mean attribute perception scores (± Standard Deviation) among participants who had not formed an opinion whether to consume fish fed with insects and ask for more information on the topic. Answers were given on a five-point Likert scale with response categories ‘1 = strongly disagree’, ‘2 = disagree’, ‘3 = neutral’, ‘4 = agree’, ‘5 = strongly agree’ (n = 46). Asterisks denote statistical differences (** *p* < 0.01, *** *p* < 0.001) after an one-sample t-test with test value = 3; n.s.— not significant.

Participants who had not formed an opinion whether to consume insect-fed fish or not reported that they needed more information about the hygiene and safety of the fish report concerns about potential allergies (Ls score = 4.20; t = 6.9, *p* < 0.001), the organoleptic characteristics of insect-fed fish (Ls score = 4.07; t = 7.8, *p* < 0.001) and its quality and nutritional value (Ls score = 3.91; t = 5.3, *p* < 0.001) (Figure 4). Finally, participants asked for more information on the extent to which the inclusion of insects in fish diets enhances and substantially helps aquaculture improve its sustainability or its environmental impact (Ls score = 3.69; t = 3.4, *p* = 0.002), but not for the fish price (Ls score = 3.16; t = 3.16, *p* = 0.117) (Figure 4).

### 3.3. Stakeholders Perspectives towards the Use of Insects in Aquafeeds

Among the companies that have completed the relevant survey questionnaire, four belong to the aquafeed sector and five to the aquaculture sector (Table 2). Some companies are new in the sector with only one year of operation, whereas others have been in business for several decades (maximum time of operation: 35 years). Variability is recorded also on the size of the participating companies ranging from small (3 employees) to large ones (with >1000 employees). The main products of the aquaculture companies are the sea bream and the sea bass, and the aquafeed companies mainly produce aquafeeds for these particular fish species. Regarding their annual production capacity, this ranges between 150–16,000 and 5500–100,000 tons for aquaculture and aquafeed companies, respectively. Most companies (7) serve the national and European market, and occasionally third countries.

All of the companies were aware of the use of insects as ingredients in aquafeeds, and six of them were favorably disposed towards this idea. Interestingly, none of the remaining companies was negative to the idea, whereas two companies were neutral and one stated that it was not properly informed on the topic. Four out of the nine companies that participated in the survey stated that they would produce or use aquafeeds based on insects. However, two of the remaining companies were neutral and two needed more information to form a clear opinion, answering “I don’t know”. Only one company out of nine stated a completely negative attitude to producing or using insect-based aquafeeds. Interestingly, the latter expressed a neutral opinion with regard to the use of insects in aquafeeds.

Among the companies that are positive to the idea of using insects meal, the most highly scored reason for this choice was the reduction on the environmental footprint of the business (4.50), followed by the development of innovation (4.25) and the enhancement of the sustainability of the business (4.00). In contrast, the effect of the use of insects on the quality of the products of the company and the production costs scored 3.00 and 2.67, respectively, indicating that these reasons do not strongly affect the decision making. Those companies which were neutral in using insects or asked for more information on the impact of the use of insect-based aquafeeds scored 5.00, 5.00, 4.75, 4.75 and 4.75, on the fish organoleptic characteristics, the fish growth and health, on the product safety, the legislative framework, and the consumers’ acceptance, respectively. The use of insects on the price of their products scored 4.25, and the extent on which the use of insects will help the companies to achieve specific goals, such as increased sustainability or reduced environmental impact scored 4.50. Finally, the main concerns of the one company which rejected the idea of using insects were the increase in the production cost due to the high price of insect meals, and the consumer acceptance.

## 4. Discussion

This study provides a first insight regarding the awareness and acceptance of insect-based feeds for aquaculture in Greece. The results of the conference survey show that the overall attitude of the majority of the participants towards the use of insects in aquafeeds is positive, as almost two thirds of them were favorable to the inclusion of insects in fish diets, and only a small percentage was opposed to this. This is also observed in the willingness of the participants to consume fish fed with insects. Similar results are reported in several recent studies investigating the public acceptance of insect-based aquafeeds [39,41,42]. For instance, in a survey on the acceptance of insects in animal feed in general conducted at a public fair in Belgium, it was reported that farmers, citizens and agriculture sector stakeholders who participated in the survey were in favor of using insects in animal feed [39]. In fact, the interviewees were reported to be positive in the cases of fish and poultry feed [39], which could be taken into account in future directions regarding legislation and regulatory aspects. Regarding the acceptance of insect-based aquafeeds specifically in aquaculture, most consumers were willing to accept the use of insects in the diet of farmed salmon in the UK [41]. Similar data have been reported for Spanish consumers in the case of farmed gilthead sea bream fed on insects [42]. High acceptance (around 90%) and willingness tο purchase and eat farmed fish fed on insect meals has been shown also for Northern-Italian consumers [40]. These results, together with the findings of the present study, point to a shift during the last years of the public’s attitude towards the use of insects in animal feeds, in view of the earlier studies that reported a hesitant attitude on this aspect. For instance, in a benchmark consumer survey conducted in 2013 as part of the EU-funded project PROteINSECT, more than half of the respondents were reported to be repulsed by the idea of consuming fish, chicken or pork fed with insects, and they had attributed this to the lack of information on this issue [47,48]. Apparently, the benefits from the use of insects in animal feed, and particularly aquafeeds, have been efficiently communicated over the last few years, contributing to the improvement of public perceptions and attitudes towards their exploitation as an alternative nutrient source for livestock, including fish farming. Yet, the data used in this study clearly suggest that the interviewees required additional information on the subject, which implies that there is not sufficient information, although insect-based aquafeed has been the focus of research for many years. Surprisingly, although the majority of the interviewees were aware of this topic, and had a good scientific and technical background on the subject, the knowledge gaps were similar to those reported above for farmers and consumers. This finding clearly underlines the urgent need for further training and knowledge transfer towards this direction.

Apart from the public and consumers’ perceptions, the perspective of the stakeholders, who implement the use of insect-based feeds in the farming practices, is of particular importance. According to the findings of this study, almost half of the feed or fish producing companies that participated in the stakeholder questionnaire were positively oriented to the use of insects in diets of farmed fish, and they confirmed their willingness to produce insect-based aquafeeds or to feed fish with diets that include insects. The companies that participated in the survey cover almost one quarter of the Greek aquaculture market and more than 75% of the Greek aquafeed market and, therefore, provide the first evidence of the overall market attitude in Greece. In a study with semi-structured interviews with key stakeholders of the Scottish Salmon farming sector, it was reported that salmon producers are not against the inclusion of insect materials in salmon diets, given that they are traceable, safe, cost-competitive and do not exert any negative impact on the fish quality [41]. The strongest acceptance of the use of insects in animal feed among stakeholders for the agriculture sector was recorded in Belgium for fish and poultry. In this case insects were perceived as a “natural” feed source [39]. In the current survey, more than one-third of the participating companies were reluctant to state that they are in favor or against the use of insects in fish diets and they highlighted the need for more information and knowledge on this topic, before they state a clear view on this issue.

The results of this study highlight the concerns of the aquaculture and aquafeed industry about the price of the insect meal. The company that stated complete disagreement with the idea of introducing insects in the production lines is among the biggest aquafeeds companies in Greece. The company pointed out that the increase in the production cost due to the high insect meal price was the main reason for the above view. Similarly, the companies that asked for more information before they decide whether to utilize insects as feed, also inquired about how the introduction of insects would affect the price of their products. Indeed, these concerns are currently realistic, as the trading price of insect meals is still high and not competitive to that of fishmeal. It is worth mentioning that an average trading price of fishmeal is about 1.5 Euro/kg, while those of *Hermetia* meal range at 3–9 Euro/kg [49]. The results of the first economic analysis on the effect of the introduction of insect meal of *T. molitor* in the diet of the European sea bass in a small fish farm in Italy showed that such a practice increases feeding, and, hence, production costs, not only due to the higher price of insect meal compared with fishmeal, but also due to the inferior feed conversion ratio achieved with insect meal [50]. Scaling-up insect production, especially if and when insects will be authorized as feed for other animals as well, such as poultry or swine, is expected to cause a reduction of the price of insects, under the baseline of intensive “insect farming” [51]. Although increased demand for insect meals may trigger its price reduction, other factors such as a potential reduced feed conversion efficiency may affect the fish growth performance and the fish production costs using insect-based aquafeeds in comparison to the commonly used fishmeal. However, a partial replacement of fishmeal by insect meals has been proved successful for almost all farmed fish species, both herbivorous/omnivorous and carnivorous, without retarding fish growth, due to their high protein contents and adequate essential amino acid profiles [14]. An important limiting factor for their use in aquafeeds is their high fat contents that, however, are characterized by low levels of polyunsaturated fatty acids, especially of EPA and DHA, which are essential to fish nutrition. It has been shown, however, that the nutritional value of insects can be improved either by a defatting process [52] or by raising them on enriched substrates in n-3 fatty acids [53,54] and, thus, could provide a better lipid quality feed for farmed fish and for the human as the consumer. The above findings are very promising towards a more environmentally friendly aquaculture but still more research is needed to enlighten the nutritional suitability of using insect meals in fish diets.

Concerns were also raised among the participants of the current survey about the effect of the insect-based diets on the organoleptic characteristics and the safety of the final fish product. Studies on the organoleptic properties of insect-fed fish are rather limited. Yet, the consumers’ beliefs and expectations concerning the taste of fish fed on insects are unfavorable [39,42], but this could be mostly attributed to a possible “prejudgment”, rather than an actual taste comparison. In contrast to the general perception, the results of the only study that included tasting trials indicates that the inclusion of insect meal in the diet of the Atlantic salmon does not significantly affect its basic organoleptic traits, such as flavor/taste, odor and texture [55]. To fill this gap, additional tasting evaluation studies are needed to acquire more knowledge on the organoleptic characteristics of farmed fish fed on insects. Further studies are also required to address the safety issues of the use of insect-based aquafeeds. For instance, although data on the chemical hazards of farmed insects are scarce, several chemical contaminants, e.g., heavy metals, can be bioaccumulated to insects, mainly through the feeding substrate used [56]. However, whether these contaminants can be further transferred to insect-fed fish is still under investigation. Recently, the occurrence of transferable antibiotic resistance genes in various species of edible insects available on the European market was reported [57,58], and the consequences of this finding for public health are far from clear and need to be further examined.

## 5. Conclusions

The results of this study show a wide awareness regarding the use of insect-based feeds in farmed fish diets among the individuals and the companies that participated in this first study undertaken in Greece. Moreover, a positive attitude towards the use of insects as feed for the rearing of farmed fish was shown for the majority of the respondents of the conference survey, as well as an increased willingness to consume farmed fish fed fish feed based on insects. This preference is mainly driven by the expectations of the respondents for the reduction of fishing pressure on wild fish stocks, the enhancement of the ecological footprint and the sustainability of aquaculture arising from the use of insects in aquafeeds. Regarding the stakeholders’ perspectives, half of the companies that participated in the survey were in favor of the inclusion of insects in fish diets and ready to incorporate this innovation into the production. The remainder urged for more information and knowledge on the implementation of the use of insects in aquaculture. However, the overall positive beliefs that are highlighted by the results of this study signal well for the future adoption of insect-based aquafeeds by the aquaculture sector in Greece.

## Figures and Tables

**Table 1:** Socio-demographic characteristics of the sample (n = 228).

Socio-Demographic Information	Frequency	% of Total
*Gender*		
Female	117	51.3
Male	111	48.7
*Age*		
≤20 years	65	28.5
21–30 years	95	41.7
31–40 years	25	11.0
41–50 years	26	11.4
≥51 years	17	7.5
Mean (SEM)		28.5 (0.8)
*Participation in HydroMediT as*		
Academic-researcher	39	17.1
Staff of the Aquaculture business	13	5.7
Staff of the Aquafeed business	10	4.4
Staff of the Public Sector	7	3.1
Student	158	69.3
No response	1	0.4
*Income*		
≤20,000$	103	45.2
20,001–30,000$	51	22.4
30,001–40,000$	28	12.3
40,001–50,000$	12	5.3
≥ 50,001$	15	6.5
No response	19	8.3
*Educational level*		
High School or equivalent	68	29.8
College or technical school	37	16.2
University degree	49	21.5
Post-graduate degree	73	32.2
No response	1	0.4

**Table 2:** Characteristics of the aquaculture and aquafeed companies that participated in the survey.

Company	Company Type	Time in Operation (Years)	Number of Employees	Main Products	Annual Capacity Production (tons)	Target Market
Company 1	Aquaculture	29	20	European sea bass (*D. labrax*) Gilthead sea bream (*S. aurata*)	300 (sea bass) 300 (sea bream)	Europe
Company 2	Aquaculture	20	550	European sea bass (*D. labrax*) Gilthead sea bream (*S. aurata*) Meagre (*Argyrosomus regius*) Red porgy (*Pagrus pagrus*)	16,000 (total)	National
Company 3	Aquaculture	31	356	European sea bass (*D. labrax*) Gilthead sea bream (*S. aurata*) Meagre (*A. regius*)	3000 (sea bass) 5500 (sea bream) 500 (meagre)	Europe Third countries
Company 4	Aquaculture	1	3	Gilthead sea bream (*S. aurata*)	150 (sea bream)	Europe
Company 5	Aquaculture	35	130	European sea bass (*D. labrax*) Gilthead sea bream (*S. aurata*)	1650 (sea bass) 450 (sea bream)	National Europe Third countries
Company 6	Aquafeed	6	4	Aquafeeds for the gilthead sea bream (*S. aurata*) and other marine species	5500 (total)	National Europe
Company 7	Aquafeed	30	>1000	Aquafeeds for several marine species	76,000 (total)	National
Company 8	Aquafeed	10	70	Aquafeeds for the European sea bass (*D. labrax*), gilthead sea bream (*S. aurata*), meagre (*A. regius*), red porgy (*P. pagrus*) and greater amberjack (*Seriola dumerili*)	100,000 (total)	National Europe
Company 9	Aquafeed	17	35	Aquafeeds for the European sea bass (*Dicentrarchus labrax*) and gilthead sea bream (*Sparus aurata*)	11,500 (for sea bass) 3500 (for sea bream)	National
